# 3-Bromo-*N*′-(2-hydroxy­benzyl­idene)benzohydrazide

**DOI:** 10.1107/S1600536808001293

**Published:** 2008-01-18

**Authors:** He-Bing Li

**Affiliations:** a Department of Chemistry and Life Sciences, Xiangnan University, Chenzhou 423000, People’s Republic of China

## Abstract

The title mol­ecule, C_14_H_11_BrN_2_O_2_, displays a *trans* configuration about the C=N and C—N bonds. The dihedral angle between the two benzene rings is 18.5 (3)°. An intra­molecular O—H⋯N hydrogen bond is observed. In the crystal structure, the mol­ecules are linked into a chain along the *c* axis by N—H⋯O and C—H⋯O hydrogen bonds.

## Related literature

For related literature, see: Ali *et al.* (2002 ▶); Allen *et al.* (1987 ▶); Cukurovali *et al.* (2002 ▶); Li (2007*a* ▶,*b* ▶); Qian *et al.* (2006 ▶); Qiu *et al.* (2006 ▶); Tarafder *et al.* (2002 ▶); Yang (2006 ▶); Yang & Guo (2006 ▶); Zhao (2006 ▶).
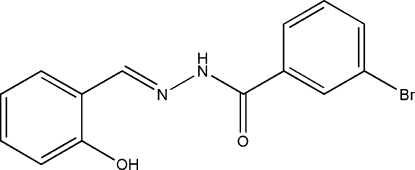


## Experimental

### 

#### Crystal data

C_14_H_11_BrN_2_O_2_
*M* *_r_* = 319.16
Monoclinic, 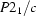

*a* = 10.9397 (17) Å
*b* = 13.672 (2) Å
*c* = 8.8915 (14) Å
β = 95.882 (2)°
*V* = 1322.8 (4) Å^3^
*Z* = 4
Mo *K*α radiation
μ = 3.11 mm^−1^
*T* = 298 (2) K
0.32 × 0.30 × 0.30 mm

#### Data collection

Bruker SMART CCD area-detector diffractometer
Absorption correction: multi-scan (*SADABS*; Sheldrick, 1996 ▶) *T*_min_ = 0.436, *T*_max_ = 0.456 (expected range = 0.377–0.394)
7853 measured reflections
3029 independent reflections
1997 reflections with *I* > 2σ(*I*)
*R*_int_ = 0.022

#### Refinement

*R*[*F* ^2^ > 2σ(*F* ^2^)] = 0.039
*wR*(*F* ^2^) = 0.106
*S* = 1.03
3029 reflections
176 parameters
1 restraint
H atoms treated by a mixture of independent and constrained refinement
Δρ_max_ = 0.73 e Å^−3^
Δρ_min_ = −0.76 e Å^−3^

### 

Data collection: *SMART* (Bruker, 1998 ▶); cell refinement: *SAINT* (Bruker, 1998 ▶); data reduction: *SAINT*; program(s) used to solve structure: *SHELXS97* (Sheldrick, 2008 ▶); program(s) used to refine structure: *SHELXL97* (Sheldrick, 2008 ▶); molecular graphics: *SHELXTL* (Sheldrick, 2008 ▶); software used to prepare material for publication: *SHELXTL*.

## Supplementary Material

Crystal structure: contains datablocks global, I. DOI: 10.1107/S1600536808001293/ci2553sup1.cif

Structure factors: contains datablocks I. DOI: 10.1107/S1600536808001293/ci2553Isup2.hkl

Additional supplementary materials:  crystallographic information; 3D view; checkCIF report

## Figures and Tables

**Table 1:** Hydrogen-bond geometry (Å, °)

*D*—H⋯*A*	*D*—H	H⋯*A*	*D*⋯*A*	*D*—H⋯*A*
O1—H1⋯N1	0.82	1.93	2.639 (3)	145
N2—H2⋯O2^i^	0.89 (1)	1.934 (15)	2.806 (3)	165 (4)
C7—H7⋯O2^i^	0.93	2.45	3.206 (3)	139

Symmetry code: (i) 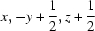.

**Fig. 1.**
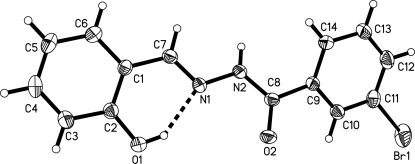
The molecular structure of the title compound, showing 30% probability displacement ellipsoids and the atom-numbering scheme. The dashed line indicates an intramolecular hydrogen bond.

**Fig. 2.**
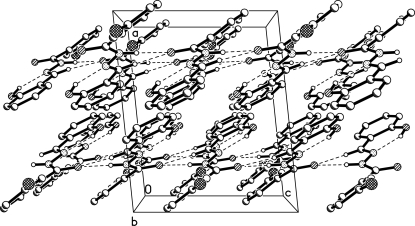
The crystal packing of the title compound, viewed along the b axis. Hydrogen bonds are shown as dashed lines.

## supplementary crystallographic information

### Comment

The compounds derived from the condensation reaction of aromatic carbaldehydes with hydrazides exhibit a wide range of biological activities and applications (Tarafder *et al.*, 2002; Cukurovali *et al.*, 2002; Ali *et al.*, 2002). Herein the author reports the crystal structure of the title compound.

The bond lengths and bond angles in the title molecule (Fig. 1) are within normal ranges (Allen *et al.*, 1987) and comprable with those observed in similar compounds(Qiu *et al.*, 2006; Yang and Guo, 2006; Yang, 2006). The C7?N1 double bond length of 1.284 (3) Å is comparable with that in other Schiff bases (Li, 2007*b*; Qian *et al.*, 2006; Zhao, 2006). The C8—N2 bond length of 1.348 (3) Å is intermediate between a C–N single bond and a C?N double bond, because of conjugation. The dihedral angle between the C1—C6 and C9—C14 benzene rings is 18.5 (3)°. The molecule adopts a *trans* configuration about the C7?N1 and C8–N2 bonds.

There is an intramolecular O1—H1···N1 hydrogen bond (Table 1) in the title molecule, as observed in a similar compound (Li, 2007*a*). In the crystal structure, the molecules are linked into a chain along the *c* axis by N—H···O and C—H···O hydrogen bonds (Table 2 and Fig.2).

### Experimental

Salicylaldehyde (0.1 mmol, 12.2 mg) and 3-bromobenzoic acid hydrazide (0.1 mmol, 21.5 mg) were dissolved in methanol (10 ml). The mixture was stirred at room temperature for 10 min to give a clear yellow solution. Crystals of the title compound were formed by gradual evaporation of the solvent over 12 d at room temperature (yield 71.2%). Analysis found: C 52.45, H 3.53, N 8.86%; calculated for C_14_H_11_BrN_2_O_2_: C 52.69, H 3.47, N 8.78%.

### Refinement

Atom H2 was located in a difference Fourier map and refined isotropically, with the N—H distance restrained to 0.90 (1) Å. The remaining H atoms were placed in geometrically idealized positions and allowed to ride on their parent atoms, with C—H = 0.93 Å, O—H = 0.82 Å, and with *U*_iso_(H) = 1.2*U*_eq_(C) and 1.5*U*_eq_(O).

### Crystal data

**Table:**

C_14_H_11_BrN_2_O_2_	*F*_000_ = 640
*M**_r_* = 319.16	*D*_x_ = 1.603 Mg m^−^^3^
Monoclinic, *P*2_1_/*c*	Mo *K*α radiation λ = 0.71073 Å
Hall symbol: -P 2ybc	Cell parameters from 2541 reflections
*a* = 10.9397 (17) Å	θ = 2.3–25.8º
*b* = 13.672 (2) Å	µ = 3.11 mm^−^^1^
*c* = 8.8915 (14) Å	*T* = 298 (2) K
β = 95.882 (2)º	Block, yellow
*V* = 1322.8 (4) Å^3^	0.32 × 0.30 × 0.30 mm
*Z* = 4	

### Data collection

**Table:**

Bruker SMART CCD area-detector diffractometer	3029 independent reflections
Radiation source: fine-focus sealed tube	1997 reflections with *I* > 2σ(*I*)
Monochromator: graphite	*R*_int_ = 0.022
*T* = 298(2) K	θ_max_ = 27.5º
ω scans	θ_min_ = 2.4º
Absorption correction: multi-scan(SADABS; Sheldrick, 1996)	*h* = −14→11
*T*_min_ = 0.436, *T*_max_ = 0.456	*k* = −17→17
7853 measured reflections	*l* = −11→11

### Refinement

**Table:**

Refinement on *F*^2^	Secondary atom site location: difference Fourier map
Least-squares matrix: full	Hydrogen site location: inferred from neighbouring sites
*R*[*F*^2^ > 2σ(*F*^2^)] = 0.039	H atoms treated by a mixture of independent and constrained refinement
*wR*(*F*^2^) = 0.106	*w* = 1/[σ^2^(*F*_o_^2^) + (0.044*P*)^2^ + 0.8938*P*] where *P* = (*F*_o_^2^ + 2*F*_c_^2^)/3
*S* = 1.03	(Δ/σ)_max_ = 0.001
3029 reflections	Δρ_max_ = 0.73 e Å^−^^3^
176 parameters	Δρ_min_ = −0.76 e Å^−^^3^
1 restraint	Extinction correction: none
Primary atom site location: structure-invariant direct methods	

### Special details

**Table:**

Geometry. All e.s.d.'s (except the e.s.d. in the dihedral angle between two l.s. planes) are estimated using the full covariance matrix. The cell e.s.d.'s are taken into account individually in the estimation of e.s.d.'s in distances, angles and torsion angles; correlations between e.s.d.'s in cell parameters are only used when they are defined by crystal symmetry. An approximate (isotropic) treatment of cell e.s.d.'s is used for estimating e.s.d.'s involving l.s. planes.
Refinement. Refinement of *F*^2^ against ALL reflections. The weighted *R*-factor *wR* and goodness of fit *S* are based on *F*^2^, conventional *R*-factors *R* are based on *F*, with *F* set to zero for negative *F*^2^. The threshold expression of *F*^2^ > σ(*F*^2^) is used only for calculating *R*-factors(gt) *etc*. and is not relevant to the choice of reflections for refinement. *R*-factors based on *F*^2^ are statistically about twice as large as those based on *F*, and *R*- factors based on ALL data will be even larger.

### Fractional atomic coordinates and isotropic or equivalent isotropic displacement parameters (Å^2^)

**Table:**

	*x*	*y*	*z*	*U*_iso_*/*U*_eq_	
Br1	0.84586 (4)	0.69664 (2)	0.07515 (6)	0.07832 (19)	
O1	0.5849 (2)	0.09555 (15)	−0.3004 (2)	0.0534 (5)	
H1	0.6305	0.1342	−0.2516	0.080*	
O2	0.7840 (2)	0.33361 (14)	−0.1658 (2)	0.0481 (5)	
N1	0.7189 (2)	0.16000 (16)	−0.0568 (2)	0.0389 (5)	
N2	0.7749 (2)	0.23255 (16)	0.0345 (3)	0.0405 (5)	
C1	0.6433 (2)	−0.00290 (19)	−0.0791 (3)	0.0368 (6)	
C2	0.5861 (3)	0.0088 (2)	−0.2268 (3)	0.0409 (6)	
C3	0.5269 (3)	−0.0700 (2)	−0.3001 (4)	0.0538 (8)	
H3	0.4882	−0.0620	−0.3974	0.065*	
C4	0.5246 (3)	−0.1593 (2)	−0.2315 (4)	0.0587 (9)	
H4	0.4853	−0.2116	−0.2831	0.070*	
C5	0.5801 (3)	−0.1726 (2)	−0.0866 (4)	0.0567 (9)	
H5	0.5784	−0.2336	−0.0405	0.068*	
C6	0.6378 (3)	−0.0954 (2)	−0.0110 (4)	0.0491 (7)	
H6	0.6740	−0.1043	0.0873	0.059*	
C7	0.7039 (3)	0.07677 (19)	0.0056 (3)	0.0398 (6)	
H7	0.7325	0.0676	0.1067	0.048*	
C8	0.8024 (3)	0.31817 (18)	−0.0293 (3)	0.0365 (6)	
C9	0.8582 (2)	0.39488 (19)	0.0755 (3)	0.0359 (6)	
C10	0.8331 (3)	0.4917 (2)	0.0361 (3)	0.0416 (7)	
H10	0.7829	0.5067	−0.0518	0.050*	
C11	0.8834 (3)	0.5656 (2)	0.1290 (4)	0.0474 (7)	
C12	0.9601 (3)	0.5450 (2)	0.2575 (4)	0.0552 (8)	
H12	0.9943	0.5954	0.3184	0.066*	
C13	0.9855 (3)	0.4493 (2)	0.2948 (3)	0.0572 (9)	
H13	1.0377	0.4349	0.3811	0.069*	
C14	0.9343 (3)	0.3738 (2)	0.2055 (3)	0.0468 (7)	
H14	0.9509	0.3091	0.2329	0.056*	
H2	0.778 (4)	0.223 (3)	0.1343 (14)	0.080*	

### Atomic displacement parameters (Å^2^)

**Table:**

	*U*^11^	*U*^22^	*U*^33^	*U*^12^	*U*^13^	*U*^23^
Br1	0.0724 (3)	0.03681 (19)	0.1239 (4)	0.00291 (16)	0.0012 (2)	−0.01230 (19)
O1	0.0719 (16)	0.0428 (11)	0.0428 (12)	−0.0081 (10)	−0.0072 (10)	0.0057 (9)
O2	0.0717 (14)	0.0422 (10)	0.0285 (11)	−0.0023 (10)	−0.0047 (9)	−0.0005 (8)
N1	0.0472 (14)	0.0350 (11)	0.0338 (12)	−0.0024 (10)	0.0004 (10)	−0.0045 (10)
N2	0.0559 (15)	0.0350 (11)	0.0292 (12)	−0.0041 (11)	−0.0022 (11)	−0.0036 (10)
C1	0.0382 (15)	0.0318 (13)	0.0409 (15)	0.0024 (11)	0.0057 (12)	−0.0011 (11)
C2	0.0441 (16)	0.0392 (14)	0.0397 (16)	−0.0023 (12)	0.0052 (12)	−0.0023 (12)
C3	0.060 (2)	0.0524 (18)	0.0481 (18)	−0.0130 (15)	−0.0005 (15)	−0.0042 (14)
C4	0.060 (2)	0.0435 (16)	0.073 (2)	−0.0143 (15)	0.0066 (18)	−0.0148 (16)
C5	0.060 (2)	0.0345 (15)	0.076 (2)	−0.0035 (14)	0.0107 (18)	0.0042 (15)
C6	0.0550 (18)	0.0398 (15)	0.0517 (18)	0.0034 (13)	0.0019 (14)	0.0053 (13)
C7	0.0452 (16)	0.0388 (14)	0.0342 (15)	0.0032 (12)	−0.0013 (12)	−0.0013 (11)
C8	0.0428 (16)	0.0359 (14)	0.0301 (15)	0.0035 (11)	0.0008 (11)	−0.0004 (11)
C9	0.0397 (15)	0.0373 (14)	0.0304 (14)	−0.0036 (11)	0.0028 (11)	−0.0023 (11)
C10	0.0437 (16)	0.0390 (15)	0.0410 (16)	−0.0004 (12)	−0.0012 (12)	−0.0042 (12)
C11	0.0468 (17)	0.0357 (14)	0.061 (2)	−0.0029 (12)	0.0098 (15)	−0.0069 (13)
C12	0.063 (2)	0.0560 (19)	0.0470 (19)	−0.0200 (16)	0.0056 (16)	−0.0149 (15)
C13	0.066 (2)	0.067 (2)	0.0364 (17)	−0.0203 (17)	−0.0083 (15)	0.0003 (15)
C14	0.0564 (19)	0.0464 (16)	0.0358 (16)	−0.0079 (14)	−0.0036 (14)	0.0043 (12)

### Geometric parameters (Å, °)

**Table:**

Br1—C11	1.889 (3)	C5—C6	1.371 (4)
O1—C2	1.354 (3)	C5—H5	0.93
O1—H1	0.82	C6—H6	0.93
O2—C8	1.228 (3)	C7—H7	0.93
N1—C7	1.284 (3)	C8—C9	1.491 (4)
N1—N2	1.384 (3)	C9—C14	1.384 (4)
N2—C8	1.348 (3)	C9—C10	1.390 (4)
N2—H2	0.89 (1)	C10—C11	1.383 (4)
C1—C2	1.404 (4)	C10—H10	0.93
C1—C6	1.406 (4)	C11—C12	1.376 (5)
C1—C7	1.446 (4)	C12—C13	1.372 (5)
C2—C3	1.385 (4)	C12—H12	0.93
C3—C4	1.367 (5)	C13—C14	1.385 (4)
C3—H3	0.93	C13—H13	0.93
C4—C5	1.379 (5)	C14—H14	0.93
C4—H4	0.93		
			
C2—O1—H1	109.5	N1—C7—H7	119.5
C7—N1—N2	116.7 (2)	C1—C7—H7	119.5
C8—N2—N1	118.6 (2)	O2—C8—N2	123.0 (2)
C8—N2—H2	124 (3)	O2—C8—C9	120.8 (2)
N1—N2—H2	117 (3)	N2—C8—C9	116.3 (2)
C2—C1—C6	118.1 (3)	C14—C9—C10	119.7 (3)
C2—C1—C7	122.5 (2)	C14—C9—C8	123.2 (2)
C6—C1—C7	119.4 (3)	C10—C9—C8	117.0 (2)
O1—C2—C3	118.2 (3)	C11—C10—C9	119.2 (3)
O1—C2—C1	122.2 (2)	C11—C10—H10	120.4
C3—C2—C1	119.5 (3)	C9—C10—H10	120.4
C4—C3—C2	121.0 (3)	C12—C11—C10	121.3 (3)
C4—C3—H3	119.5	C12—C11—Br1	120.1 (2)
C2—C3—H3	119.5	C10—C11—Br1	118.6 (2)
C3—C4—C5	120.5 (3)	C13—C12—C11	119.1 (3)
C3—C4—H4	119.7	C13—C12—H12	120.5
C5—C4—H4	119.7	C11—C12—H12	120.5
C6—C5—C4	119.5 (3)	C12—C13—C14	120.9 (3)
C6—C5—H5	120.2	C12—C13—H13	119.6
C4—C5—H5	120.2	C14—C13—H13	119.6
C5—C6—C1	121.3 (3)	C9—C14—C13	119.8 (3)
C5—C6—H6	119.3	C9—C14—H14	120.1
C1—C6—H6	119.3	C13—C14—H14	120.1
N1—C7—C1	121.0 (2)		

### Hydrogen-bond geometry (Å, °)

**Table:**

*D*—H···*A*	*D*—H	H···*A*	*D*···*A*	*D*—H···*A*
O1—H1···N1	0.82	1.93	2.639 (3)	145
N2—H2···O2^i^	0.89 (1)	1.934 (15)	2.806 (3)	165 (4)
C7—H7···O2^i^	0.93	2.45	3.206 (3)	139

Symmetry codes: (i) *x*, −*y*+1/2, *z*+1/2.
